# Individuals with High Mindfulness Are Better at Metacognitive Ability: A Latent Profile Analysis Approach

**DOI:** 10.3390/bs15101341

**Published:** 2025-09-29

**Authors:** Lan Jiang, Shang Zhang, Jinglin Li, Yuhong Gong, Na Sun, Haihong Wang, Tao Xiao, Xinfa Yi

**Affiliations:** 1 College of Humanities & Arts, Xi’an International University, Xi’an 710077, China; jianglan0327@sina.com; 2 Key Laboratory of Modern Teaching Technology (Ministry of Education), Shaanxi Normal University, No. 199 Chang’an Road, Yanta District, Xi’an 710062, China; lijinglin@snnu.edu.cn; 3 Faculty of Education, Shaanxi Normal University, No. 199 Chang’an Road, Yanta District, Xi’an 710062, China; zhang_shang2003@163.com (S.Z.); gongyh2024@163.com (Y.G.); sunwange@snnu.edu.cn (N.S.); wanghaihong@nwu.edu.cn (H.W.); 4 School of Physical Education, Shaanxi Normal University, 620 West Chang’an Avenue, Chang’an District, Xi’an 710062, China

**Keywords:** mindfulness, metacognition, latent profile analysis, highly mindful

## Abstract

This study systematically examines the relationship between mindfulness and metacognition among Chinese college students through a person-centered analytical approach. Using latent profile analysis (LPA) of Five Facet Mindfulness Questionnaire (FFMQ) responses, we identified four distinct mindfulness profiles: (1) High Observation/Low Non-reactivity, (2) High Awareness/Judging, (3) Moderately Mindful, and (4) Highly Mindful. Gender differences were observed across profiles, with female students more represented in the Highly Mindful group. Hierarchical regression analyses revealed that mindfulness profiles significantly predicted metacognitive ability, with the Highly Mindful group demonstrating superior metacognitive self-regulation and learning strategy application. These findings contribute to the literature by identifying distinct mindfulness subtypes and their differential relationships with metacognition. The results suggest that educational interventions emphasizing non-judgmental present-moment awareness may be particularly effective for fostering students’ metacognitive development, while highlighting the importance of considering individual differences in mindfulness training approaches.

## 1. Introduction

Mindfulness, originating from Buddhism, is a psychological practice and philosophical concept centered around awareness and attention (Kabat-Zinn, 2003). It entails cultivating a clear, unbiased awareness and experience of the present moment, emphasizing principles of listening and mindfulness as foundational principles (Ford et al., 2020). Mindfulness has been extensively studied in psychology and is widely utilized as a psychological intervention method. Recent meta-analytical studies from various perspectives have confirmed the positive effects of mindfulness on self-awareness, mental and physical health, emotional regulation, and stress (Fendel et al., 2020; Hamasaki, 2023; Jawad et al., 2023; Michaelsen et al., 2023; Sharpe et al., 2024; G. Wang et al., 2023). Research indicates that mindfulness helps individuals reconnect with the present moment, understand their emotions, listen attentively to others, and improve interpersonal relationships and mental well-being (Burgos et al., 2022; Hamasaki, 2023).

To measure mindfulness from different perspectives, researchers have developed various assessment tools. Among these, the Five Facet Mindfulness Questionnaire is one of the most commonly used self-report measures (Lecuona et al., 2022). These five facets are Observing, Describing, Acting with Awareness, Non-judging, and Non-reactivity (Baer et al., 2006). Observing involves paying attention to internal and external experiences without judgment, including breath, bodily sensations, emotions, and thoughts. Describing refers to an individual’s ability to verbalize and articulate their experiences, promoting better understanding and processing of inner experiences. Acting with Awareness emphasizes an individual’s self-awareness and self-attention during actions. It requires individuals to be attentive to the current task or experience during their actions, rather than acting absentmindedly or aimlessly. Non-judging encourages individuals to maintain a non-judgmental and non-critical attitude towards their internal perceptions, emotions, and thoughts. This means not assigning value judgments, such as “good” or “bad,” to their inner experiences. Non-reactivity involves an individual’s tendency not to react immediately or become emotionally agitated. It encourages individuals to remain calm and composed when facing inner experiences, without being emotionally manipulated or overreacting. These dimensions of the Five Facet Mindfulness Questionnaire are core elements of mindfulness training and contribute to enhancing an individual’s awareness and coping abilities concerning internal and external experiences (Ford et al., 2020; Li Anthony et al., 2023).

Historically, research has primarily explored the effects and mechanisms of mindfulness through variable-centered and individual-centered pathways (Borchers & Pieler, 2010). While variable-centered research methods have been dominant, their practicality and the low intercorrelations among different facets of mindfulness limit their effectiveness in guiding mindfulness practice (Cheli et al., 2019). For example, when using variable-centered methods to analyze the Five Facet Mindfulness Questionnaire (FFMQ), each facet is treated as a separate measurement unit, examining its unique effects on specific outcome variables (Li Anthony et al., 2023). This limitation in the approach results in limited information and clues, as analyzing individual scores on a single dimension cannot address complex interactions within the multidimensional nature of mindfulness (Lam et al., 2018). As a consequence, this approach is inadequate for exploring the intricate interplay of multiple dimensions of mindfulness.

To overcome the limitations of variable-centered research methods, researchers have turned their attention to individual-centered approaches in recent years (Bravo et al., 2016; Sahdra et al., 2023). Individual-centered research methods, such as mean-splitting, cluster analysis, latent profile analysis, and others. These methods are employed to examine response patterns on various items for each study participant. By extracting those participants with similar response patterns, subgroups can be formed, allowing for the identification of heterogeneous groups (De Souza Marcovski & Miller, 2023). Since individual-centered research methods no longer focus solely on the examination of specific variables, they are better suited to reflect the comprehensive characteristics of individuals (Ford et al., 2020).

Currently, Latent Profile Analysis (LPA) is commonly used to explore profiles of the FFMQ among different groups and examine their impact on various outcome variables (Yalçın et al., 2022). In fact, the purpose of conducting latent profile analysis on mindfulness is to uncover its latent categories, treating mindfulness as a holistic system that takes into account the interactions between different facets of the FFMQ. It aims to understand the internal mechanisms and components of mindfulness by grouping study participants into different subgroups (profiles) based on similar dimensions, and subsequently analyzing the differences among these subgroups on outcome variables (Li Anthony et al., 2023). Specifically, Latent Profile Analysis is a multivariate data analysis method that transforms observational data into an internal structural model, offering a more detailed and comprehensive perspective to comprehend concepts that are challenging to perceive (Sahdra et al., 2023). In summary, the essential logic behind employing latent profile analysis on mindfulness is to help us gain a more comprehensive understanding of the essence and practice of mindfulness by exploring its internal structure and constituents. This method effectively addresses the limitations of variable-centered approaches, providing valuable guidance for the development and enhancement of mindfulness practices.

Prior research employing latent profile analysis on the FFMQ across different participant groups has revealed variations in the latent types of mindfulness among different age groups and cultural backgrounds. Pearson et al. (2015) conducted profile analysis on the mindfulness of 941 American university students, resulting in four latent categories. These categories include the low-mindfulness class (characterized by low scores across all aspects of mindfulness, 59% of the sample), the high-mindfulness class (with relatively high scores or medium scores across all facets of mindfulness, 26%), the judgmental observing group (highest in observing but very low in non-judging, 6%), and the non-judgmental aware group (characterized by a high degree of non-judgment towards inner experiences and acting with awareness but very low in observing, 7%). Among these profiles, only college students, with a mean age of 20, participated in this study. The generalizability of these groups remained unaddressed.

Subsequent studies have replicated these four types (Bravo et al., 2016; Lam et al., 2018). These studies also exhibited similar proportions in sample sizes. Specifically, the low-mindfulness class had the largest sample size proportion, followed by the high-mindfulness class, and finally, the judgmental observing and non-judgmental aware groups. Sahdra et al. (2023) expanded the sample size and the age range of participants. Their research identified two additional unique characteristics: moderate mindfulness and moderately non-judgmental feature groups. De Souza Marcovski and Miller (2023) further extended the age range of participants from 18 to 75 years and almost replicated the earlier four-category findings. However, in their study, the mid-mindfulness group (37.3%) replaced the low-mindfulness group, and it had the highest proportion of individuals in the sample (De Souza Marcovski & Miller, 2023). These results have been replicated across different populations, including video game players, samples with a history of recurrent depression, and the adult population in Spain, among others (Carlon et al., 2023; Gu et al., 2017; Lecuona et al., 2022; Li Anthony et al., 2023).

Combining findings from previous research, the results of profile analysis on mindfulness in adult participant groups exhibit a certain degree of stability, but occasional inconsistencies also exist. These inconsistencies may be related to factors such as the age range and cultural background of the participants (Ford et al., 2020). Therefore, it is crucial to verify the previous results in different age groups or diverse community samples. Further examination of mindfulness profiles in a wide range of samples may help address these inconsistencies. Given the relative scarcity of studies exploring the latent types of mindfulness among Chinese mainland university students, this study aims to investigate the latent types of mindfulness among Chinese university students using latent profile analysis, building upon existing theories and empirical research. The goal is to understand the characteristics of mindfulness among this population, providing a basis for more precise and efficient differentiated mindfulness interventions. Based on this, we propose the following hypotheses:

**H1.**  *Chinese university students exhibit significantly heterogeneous mindfulness profiles. It is anticipated that latent profile analysis will identify 3–5 distinct mindfulness subtypes, whose classification characteristics will align with previously identified international profile structures while also potentially demonstrating culturally specific patterns due to contextual differences*.

Mindfulness, derived from various traditional Buddhist contemplative practices, plays a pivotal role in enhancing individual awareness and self-perception, improving emotional regulation, increasing self-awareness, and augmenting metacognitive skills (Jawad et al., 2023). As a result, the relationship between mindfulness and metacognition has garnered increasing attention. Metacognition refers to individuals’ perception and understanding of how they learn, remember, solve problems, and control cognitive activities (Flavell, 1979). It is the “cognition about cognition,” involving individuals’ awareness and understanding of their cognitive and learning activities, pre-planning for cognitive activities, monitoring during thinking processes, and evaluation during or after tasks (Efklides, 2002; Flavell, 1979). Metacognition plays a crucial role in learning, problem-solving, decision-making, and task execution (Csikos, 2022). Individuals with high metacognitive abilities can more effectively manage their cognitive resources, enhance learning efficiency, better solve problems, and make wiser decisions (Williams et al., 2016). Metacognition is also an important area of research in education and psychology, contributing to the understanding of how people learn and think.

Previous research has employed a variable-centered approach to theoretically and empirically investigate and confirm the relationship between mindfulness and metacognition. For example, the Cognitive Control Theory suggests that mindfulness practice can enhance self-monitoring and self-control abilities, thus improving metacognition (J. R. Schmidt et al., 2023). Specifically, mindfulness can help individuals gain a clearer understanding of their cognitive processes and better control their thoughts and emotions, enabling more effective management of cognitive tasks, learning, and problem-solving (Baird et al., 2014; C. Schmidt et al., 2019). The Self-Regulation Model posits that mindfulness, by enhancing metacognitive guidance, influences how individuals regulate their thoughts and emotions by monitoring and guiding themselves (Razavizadeh Tabadkan & Mohammadi Poor, 2016). This implies that through mindfulness practice, individuals can better understand and manage their thought and emotional responses, thereby improving the quality and effectiveness of thought and emotion regulation. This theory emphasizes the potential of mindfulness as a self-regulation tool, and self-regulation and control are core components of metacognition. The Attentional Theory suggests an inherent connection between mindfulness and attention. They can influence metacognition through similar attention control processes (Aldahadha, 2020; Bokk & Forster, 2022; Razavizadeh Tabadkan & Mohammadi Poor, 2016). This means that by cultivating focus and mindfulness, individuals can enhance their metacognition, which involves monitoring and adjusting their cognitive processes (Solem et al., 2017).

Furthermore, empirical research has conducted some confirmatory work based on the theories mentioned earlier. Cross-sectional studies have confirmed the positive effects of mindfulness on metacognition (Deng et al., 2019). Research has found that enhancing mindfulness may help reduce mind-wandering and is associated with better metacognition (Levinson et al., 2014). A four-week breath counting mindfulness training can reduce mind-wandering and, in turn, improve metacognition (Deng et al., 2019; Levinson et al., 2014). Additionally, it has been found that higher levels of mindfulness are related to less dysfunctional metacognition, indicating that mindfulness may aid in managing metacognition and reducing the chances of depressive symptoms and recurrence (Aldahadha, 2020). This includes the management of negative evaluations of internal thoughts and negative thoughts like uncontrollable rumination (Solem et al., 2017). Research has also shown that mindfulness has a positive impact on improving aspects of metacognition such as motor control, cognitive flexibility, and executive functions in individuals with metacognitive impairments (Aldahadha, 2020).

In summary, these studies suggest that metacognition and mindfulness are two related but independent constructs (Norman et al., 2019), and mindfulness training affects metacognition by enhancing individuals’ self-awareness, emotional regulation, and thought regulation abilities.

However, not all studies support a positive relationship between these two. C. Schmidt et al. (2019) assessed participants’ metacognitive efficiency before and after engaging in two different types of meditation training programs. They found that the metacognitive efficiency of the group practicing psychological cues remained stable, while the group practicing bodily cues experienced a significant decrease in metacognitive efficiency. This suggests that the relationship between mindfulness and metacognition might be influenced by the specific mindfulness practices and content (C. Schmidt et al., 2019). Subsequently, Böckler and Singer (2022) conducted a 9-month psychological training program involving three different modules and found that metacognition did not change significantly in the participants following the training.

It is evident that mindfulness intervention research faces various challenges, with many variables centered studies offering limited clues for more precise interventions. Currently, there is a lack of research exploring the impact of mindfulness types on metacognition and its practical applications based on person-centered methods. Given this, the present study, drawing upon previous theories and empirical research, employs latent profile analysis to investigate the latent types of mindfulness in Chinese university students and their effects on metacognition. This provides a new research perspective for understanding metacognitive differences from the viewpoint of mindfulness types.

The current body of research lacks comprehensive analysis on how different types of mindfulness influence metacognition and their practical implications when using person-centered methodologies. Addressing this gap, our study adopts a person-centered approach to investigate the distinct mindfulness characteristics in college students and examine their association with metacognition. Based on this, we propose the following hypotheses:

**H2.**  *Significant differences exist in total metacognition scores and subdimensions among different mindfulness profile groups. Specifically, the high mindfulness group demonstrates significantly superior performance in both overall metacognition and specific subdimensions—including planning, monitoring, and regulation—compared to other mindfulness groups*.

## 2. Materials and Methods

### 2.1. Participants

The study employed an online convenience sampling method to recruit participants from a comprehensive university in China. A total of 710 undergraduate students initially participated in the survey. Following rigorous data screening procedures that excluded responses with incomplete or inconsistent patterns (e.g., straight-lining or random responding), the final analytical sample comprised 689 valid cases (226 male, 463 female), yielding an effective response rate of 97.04%. Participants’ ages ranged from 18 to 23 years (M = 20.38, SD = 0.89), representing typical undergraduate cohorts in Chinese higher education.

Academic discipline distribution was as follows: humanities (45.32%, *n* = 312), natural sciences (43.93%, *n* = 303), engineering (6.54%, *n* = 45), and arts disciplines (4.21%, *n* = 29), with the latter category encompassing music, sports, and visual arts. This distribution approximates the institutional demographics of the source population.

Prior to data collection, all participants provided written informed consent through a digital consent form that detailed study procedures, data confidentiality protocols, and voluntary participation rights. The research protocol received formal ethical approval from the Institutional Review Board at the Key Laboratory of Modern Teaching Technology, Ministry of Education, Shaanxi Normal University (IRB Approval No. L20230711-01; Date: 30 September 2022), ensuring compliance with the Declaration of Helsinki guidelines for human subjects research.

### 2.2. Five Facet Mindfulness Questionnaire (FFMQ)

The mindfulness assessment employed the Five Facet Mindfulness Questionnaire (FFMQ), originally developed by Baer et al. (2006) and subsequently validated in Chinese populations by Deng et al. (2011). This well-established instrument measures five distinct facets of mindfulness: (1) Observing (noticing internal and external experiences), (2) Describing (verbalizing experiences), (3) Acting with Awareness (present-moment attention), (4) non-judging of inner experience (acceptance of thoughts/feelings), and (5) Non-reactivity to inner experience (allowing thoughts/feelings to come and go).

The 39-item scale utilizes a 5-point Likert-type response format ranging from 1 (“not at all true of me”) to 5 (“very often true of me”), with higher composite scores indicating greater mindfulness capacity. In the current sample, the measure demonstrated marginally acceptable internal consistency (Cronbach’s α = 0.73), which falls within the lower range of reliability coefficients reported in previous validation studies with Chinese student populations (Deng et al., 2011; Chen et al., 2020).

### 2.3. College Student Metacognitive Ability Scale (CSMAS)

The metacognitive evaluation employed the College Student Metacognitive Ability Scale (CSMAS; Jiang et al., 2023), a 24-item instrument assessing four dimensions on a 5-point Likert scale (1 = “never” to 5 = “always”): (1) metacognitive planning (7 items; e.g., “I understand when and why to use different learning strategies”), (2) metacognitive monitoring (6 items; e.g., “I check if I have achieved my learning objectives during the process”), (3) metacognitive adjustment (6 items; e.g., “I change my learning methods to adapt to different requirements”), and (4) metacognitive evaluation (5 items; e.g., “After completing a task, I reflect on whether there was an easier approach”).

A total metacognitive ability score was computed by summing responses across all subscales. The scale demonstrated excellent internal consistency in our sample (Cronbach’s α = 0.85), consistent with previous validations (Sun & Tian, 2015; S. Wang et al., 2022).

### 2.4. Data Analysis

The current investigation employed a comprehensive analytical approach utilizing specialized statistical software packages. For preliminary data screening and fundamental analyses, SPSS Statistics version 24.0 was implemented to conduct: (1) Harman’s single-factor test to assess potential common method variance; (2) descriptive statistical analyses including means, standard deviations, and distribution characteristics; and (3) bivariate Pearson correlation analyses to examine interrelationships among key study variables.

For advanced modeling procedures, Mplus version 7.4 was utilized to perform latent profile analysis (LPA), a person-centered analytical technique particularly suited for identifying homogeneous subgroups within heterogeneous populations. The LPA procedure incorporated (a) iterative model fitting with increasing profile solutions, (b) evaluation of multiple fit indices (AIC, BIC, aBIC, BLRT, and LMR), and (c) substantive interpretation of emergent profiles.

Subsequent between-profile comparisons were conducted using the BCH method (Bolck et al., 2004), a robust three-step approach that (1) estimates latent class probabilities, (2) assigns individuals to classes based on maximum probability, and (3) tests mean differences across distal outcomes while accounting for classification uncertainty. This method has been demonstrated to yield unbiased estimates in simulation studies (Asparouhov & Muthén, 2014; Bakk & Vermunt, 2016).

## 3. Results

### 3.1. Test of Common Method Bias

To address potential common method bias (CMB) inherent in our cross-sectional design with single-time-point self-reported measures, we employed Harman’s single-factor test (Podsakoff & Organ, 1986). Principal component analysis without rotation yielded 11 factors with eigenvalues exceeding 1.0, collectively explaining only 23.11% of the total variance—substantially below the recommended 40% threshold for significant CMB (Podsakoff et al., 2003). These results suggest that common method variance does not pose a substantial threat to the validity of our findings.

### 3.2. Descriptive Statistics of Key Variables

The correlation analysis revealed significant positive associations between metacognition and the mindfulness facets of observing, describing, acting with awareness, and non-reactivity. In contrast, a significant negative correlation was observed between metacognition and the non-judging facet (see Table 1).

### 3.3. Latent Profile Analysis Results

The current study employed latent profile analysis (LPA) in Mplus 7.4 to identify distinct mindfulness profiles among college students. Beginning with a two-profile solution, we systematically compared models with incrementally increasing numbers of latent profiles to determine the optimal classification based on established fit criteria (Nylund-Gibson et al., 2007). Model selection was guided by multiple indicators: lower values of Akaike’s Information Criterion (AIC), Bayesian Information Criterion (BIC), and sample-size-adjusted BIC (SSA-BIC) signified better model fit; entropy values exceeding 0.8 indicated satisfactory classification accuracy; significant results (*p* < 0.05) from both the Lo–Mendell–Rubin Likelihood Ratio Test (LMR-LRT) and Bootstrap Likelihood Ratio Test (BLRT) suggested meaningful model improvement; and each profile’s estimated class probability was required to exceed 5% of the sample.

As presented in Table 2, the likelihood ratio tests indicated statistically significant improvements in model fit for the two-class, three-class, and four-class solutions (all LMR-LRT and BLRT *p* < 0.05). In contrast, the five-class solution did not yield a significant improvement in fit over the four-class model, suggesting that the four-class solution represents the optimal profile structure. Comparative evaluation of the viable models revealed that the four-class solution showed superior fit, as evidenced by the lowest values across all information criteria (AIC, BIC, and SSA-BIC), coupled with the highest entropy value, reflecting optimal classification precision. These results collectively supported the selection of the four-profile model as the most parsimonious and psychologically meaningful solution, effectively distinguishing four distinct mindfulness subtypes within the college student population. The identified profiles demonstrated both statistical robustness and theoretical relevance, suggesting they represent authentic patterns of mindfulness characteristics rather than artificial statistical artifacts.

The Five Facet Mindfulness Questionnaire (FFMQ) was administered using a 5-point Likert scale (range: 1–5), with a theoretical midpoint of 3. Following Bravo et al. (2016), scores below 2.30 were classified as low, above 3.70 as high, and intermediate scores as moderate.

**Figure 1:**
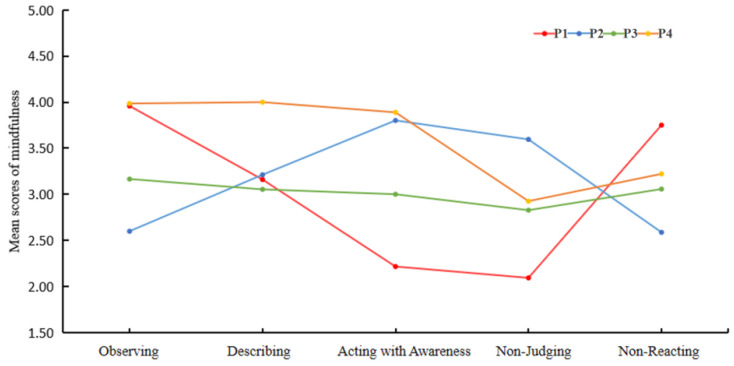
Latent profile plot of mindfulness. Note: In Figure 1, P1 represents the High Observation/Low Non-reactivity group, P2 represents the High Awareness/Judging group, P3 represents the Moderately Mindful group, and P4 represents the Highly Mindful group.

Latent profile analysis revealed four distinct mindfulness profiles, each reflecting unique patterns across the five facets of mindfulness. The largest profile, termed the Moderately Mindful group (54.57%), exhibited scores near the scale midpoint on all dimensions, suggesting a balanced but moderate engagement with mindfulness practices. The second profile, labeled the High Observation/Low non-judging group (18.58%), demonstrated a pronounced dissociation between facets, with the highest Observing scores (3.96) coupled with the lowest non-judging scores (2.09), a pattern consistent with prior reports of individuals who actively notice experiences but struggle to accept them without evaluation. In contrast, the High Awareness/Judging group (16.84%) showed elevated Acting with Awareness (3.80) and non-judging (3.60) scores but relatively low Observing scores (2.60), indicating a profile characterized by present-moment engagement rather than detached observation. Finally, the Highly Mindful group (10.01%) displayed uniformly high scores across all facets, aligning with theoretical models of integrative mindfulness. Complete statistical results, including fit indices and profile comparisons, are presented in Table 3 and visualized in Figure 1.

### 3.4. Relationship Between Demographic Variables and Latent Types of Mindfulness

The analysis revealed significant gender differences in mindfulness profile membership, with females showing a higher likelihood of belonging to certain profiles, while age demonstrated no significant associations. Odds ratios (OR) were computed to quantify the relative probability of profile membership between comparison groups. Specifically, females had 3.636 times higher odds (OR = 3.64, 95% CI [1.12, 5.15]) of being classified in the High Awareness/Judging group (P2) compared to the Observing group (P1) relative to males. Further comparisons indicated that females were significantly more likely to belong to the High Awareness/Judging group (P2) than both the Observing group (P1) and the Moderately Mindful group (P3), with no other significant inter-group differences observed. These findings suggest distinct gender-based patterns in mindfulness profile distribution, particularly highlighting women’s greater propensity for the High Awareness/Judging group orientation characterized by P2.

### 3.5. Effects of Mindfulness Profiles on Metacognitive Functioning

The analysis revealed significant variations in metacognitive functioning across the four identified mindfulness profiles, with distinct patterns emerging across planning (*χ*^2^ = 129.34, *p* < 0.001), monitoring (*χ*^2^ = 126.97, *p* < 0.001), regulation (*χ*^2^ = 117.91, *p* < 0.001), and evaluation (*χ*^2^ = 136.49, *p* < 0.001) domains (Table 4). Post hoc comparisons with Bonferroni adjustment demonstrated a clear hierarchical structure in metacognitive performance. The Highly Mindful group exhibited consistently higher performance across all metacognitive dimensions compared to other profiles (all *p* < 0.001, Cohen’s *d* = 0.82–1.15), followed by the High Observation/Low Non-reactivity who showed intermediate levels of metacognitive functioning that were significantly higher than both the High Awareness/Judging group and Moderately Mindful groups (*p* < 0.001, *d* = 0.45–0.62). Notably, no significant differences were observed between the High Awareness/Judging and Moderately Mindful groups on any metacognitive measures (*p* > 0.05, *d* < 0.20). These patterns indicate a graded relationship between mindfulness profile characteristics and metacognitive abilities, where more integrated mindfulness profiles—particularly those characterized by balanced awareness and emotional non-reactivity—tend to co-occur with enhanced metacognitive regulation. They are consistent with theoretical models that posit mindfulness as a foundational component of metacognitive self-regulation in emerging adults, potentially informing targeted mindfulness-based interventions in educational settings. The large effect sizes observed between profile groups highlight the clinical significance of these dispositional differences in cognitive functioning.

## 4. Discussion

Adopting an individual-centered research perspective, this study utilized latent profile analysis to examine whether Chinese university students exhibit distinct latent categories of mindfulness and whether these categories differ with respect to metacognitive planning, monitoring, regulation, and evaluation. The findings reveal four distinct latent mindfulness profiles among Chinese university students. Specifically, the profiles were classified as follows: (1) High Observation/Low Non-reactivity group (characterized by strong observational skills but significantly lower scores in non-reactivity to inner experiences, 18.58% of the sample), (2) High Awareness/Judging group (exhibiting elevated scores in awareness/judging alongside average observation scores, 16.84%), (3) Moderately Mindful group (demonstrating moderate levels across all five facets, 54.57%), and (4) Highly Mindful group (showing elevated scores across all five mindfulness facets, 10.01%).

Overall, this study has successfully validated the existence of four mindfulness profiles in the Chinese sample, thereby confirming Hypothesis 1, while replicating and extending previous research findings (Bravo et al., 2016; Pearson et al., 2015; Lam et al., 2018). Simultaneously, the results reveal significant differences in the distribution patterns among these four mindfulness profiles. Specifically, the “Moderately Mindful” profile was most prevalent among Chinese student samples (reaching 54.57%)—a distribution characteristic that stands in sharp contrast to Western populations where the “High Mindfulness” or “Low Mindfulness” profiles typically dominate (Bravo et al., 2016; Pearson et al., 2015; Lam et al., 2018). These distributional differences not only strengthen empirical support for the cross-cultural universality of mindfulness profiles, but also highlight the uniqueness within the Chinese cultural context. The Chinese cultural emphasis on emotional moderation and behavioral restraint may be closely related to this pattern, as such cultural environments likely foster more balanced and temperate expressions of mindfulness traits.

The current findings reveal significant gender differences in mindfulness profile distribution, with female students substantially overrepresented in the High Awareness/Judging group compared to their male counterparts. This pattern aligns with recent longitudinal evidence highlighting gender-divergent pathways in mindfulness development (Zhang et al., 2024), suggesting that such disparities may reflect deeper sociocognitive trends. A plausible explanation for this phenomenon may lie in gender role socialization, which shapes how individuals express mindfulness through culturally reinforced traits (Stoltzfus et al., 2011). Traditional feminine roles, which often emphasize emotional attunement and interpersonal sensitivity, may foster the specific cognitive and affective skills captured in the High Awareness/Judging profile—such as heightened self-observation and a tendency toward evaluative awareness. Thus, the elevated representation of women in this profile may not merely indicate a psychological difference, but also reflect the broader influence of socially constructed gender norms.

College students belonging to different latent mindfulness categories exhibit significant differences in metacognitive planning, monitoring, regulation, and evaluation. These results confirm Hypothesis 2. Specifically, the Highly Mindful group scores significantly higher in all metacognitive dimensions compared to the other three groups. The High Observation/Low Non-reactivity group scores significantly higher than the High Awareness/Judging group and the Moderately Mindful group in all metacognitive dimensions. However, there is no significant difference in scores between the High Awareness/Judging group and the Moderately Mindful group. These results indicate that college students from different mindfulness categories exhibit variations in their metacognitive levels. This suggests a relationship between college students’ metacognitive abilities and mindfulness (C. Schmidt et al., 2019; Aldahadha, 2020). Therefore, this information can be used to guide college students in effectively enhancing their metacognitive abilities through targeted mindfulness interventions, taking into account the characteristics and heterogeneity of different mindfulness groups, thus achieving precision intervention.

Specifically, the Highly Mindful group scores the highest in all metacognitive dimensions, which is consistent with previous research indicating that enhanced mindfulness is beneficial for improving metacognition (Levinson et al., 2014). This can be explained by the cognitive control theory. Individuals with high levels of mindfulness can more clearly observe and describe their cognitive processes, consciously regulate their thoughts and emotions, and effectively manage cognitive tasks, learning, and problem-solving (J. R. Schmidt et al., 2023). The High Observation/Low Non-reactivity group scores significantly higher in all metacognitive dimensions compared to the High Awareness/Judging group and the Moderately Mindful group. However, there is no significant difference in scores between the High Awareness/Judging group and the Moderately Mindful group. Analyzing the characteristics of these three categories reveals that the High Observation/Low Non-reactivity group has the strongest observation abilities, while the middle mindfulness group and the High Awareness/Judging group exhibit average or weaker observational skills.

This reflects the importance of training in a particular aspect, such as observation. Observation is a manifestation of focused behavior, where individuals pay attention and become aware of their internal and external perceptions and experiences without critical judgment. This includes focusing on aspects like breathing, bodily sensations, emotions, thoughts, and doing so with non-judgmental awareness (Aldahadha, 2020; Bokk & Forster, 2022). This aligns with the emphasis of mindfulness on the connection between mindfulness and concentration, as they both enhance an individual’s metacognition through similar attention control processes, specifically in monitoring and adjusting one’s cognitive processes (Solem et al., 2017; Verhaeghen, 2020). In summary, these research results support the idea that metacognition and mindfulness are two related yet independent constructs (Norman et al., 2019). Mindfulness training improves metacognition by enhancing individuals’ self-awareness, emotional regulation, and thought regulation abilities.

## 5. Limitations and Conclusions

This study identified distinct mindfulness profiles among Chinese college students, demonstrating that metacognitive functioning varies meaningfully across these subgroups. The findings challenge the “more is better” assumption, revealing that specific facet configurations—particularly strong observation skills—are most conducive to metacognitive enhancement.

However, these conclusions should be interpreted in light of several methodological considerations. First, the study’s reliance on self-report measures may introduce response biases, and future research should incorporate multimodal assessments (e.g., behavioral tasks, physiological indices, or neuroimaging) to enhance objectivity. Second, the cross-sectional design limits causal inference regarding mindfulness development; longitudinal and experience-sampling approaches are needed to examine how these profiles evolve over time. Third, the cultural and demographic specificity (Chinese university students) calls for replication in broader populations to assess generalizability.

Despite these constraints, the findings highlight the value of person-centered approaches in capturing mindfulness heterogeneity. Rather than assuming uniform benefits, tailored interventions—particularly those emphasizing observation training—may optimize metacognitive outcomes. Future studies should test these hypotheses through experimental and longitudinal designs while integrating neuroscientific and behavioral measures to advance a more nuanced understanding of mindfulness as a dynamic self-regulatory process.

## Figures and Tables

**Table 1:** Key variable means, standard deviations, and correlations (*n* = 689).

	M	SD	1	2	3	4	5	6	7	8
1 Observing	3.30	0.62	1.00							
2 Describing	3.20	0.55	0.29 **	1.00						
3 Acting with Awareness	3.08	0.68	−0.33 **	0.28 **	1.00					
4 Non-judging	2.83	0.60	−0.52 **	0.02	0.58 **	1.00				
5 Non-reacting	3.12	0.54	0.55 **	0.12 **	−0.47 **	−0.56 **	1.00			
6 Planning	3.61	0.63	0.32 **	0.28 **	0.17 **	−0.13 **	0.26 **	1.00		
7 Monitoring	3.57	0.68	0.33 **	0.30 **	0.15 **	−0.17 **	0.25 **	0.81 **	1.00	
8 Adjustment	3.71	0.58	0.30 **	0.29 **	0.19 **	−0.13 **	0.24 **	0.85 **	0.73 **	1.00
9 Evaluation	3.75	0.64	0.33 **	0.28 **	0.15 **	−0.19 **	0.27 **	0.816 **	0.82 **	0.81 **

Note: ** *p* < 0.01.

**Table 2:** Latent profile analysis results for the five facets of mindfulness.

Model	k	BIC	aBIC	Entropy	LMR (*p*)	BLRT (*p*)	Category Probabilities
2C	16	5751.70	5700.90	0.76	<0.001	<0.001	0.695/0.305
3C	22	5615.75	5545.89	0.82	<0.001	<0.001	0.296/0.617/0.087
4C	28	5400.23	5311.33	0.82	0.002	<0.001	0.186/0.163/0.546/0.100
5C	34	5390.70	5282.74	0.84	1.000	<0.001	0.158/0.187/0.553/0.029/0.073

**Table 3:** Number, proportion, mean scores, and standard deviations of different profiles of Five Facet Mindfulness Questionnaire (M ± SD).

Latent Categories	*n*	Proportion	Observing	Describing	Acting with Awareness	Non-Judging	Non-Reacting
H/L	128	18.58%	3.96 (0.46)	3.16 (0.58)	2.22 (0.45)	2.09 (0.41)	3.75 (0.48)
A/J	116	16.84%	2.60 (0.45)	3.21 (0.54)	3.80 (0.36)	3.60 (0.41)	2.59 (0.42)
MM	376	54.57%	3.17 (0.37)	3.05 (0.42)	3.00 (0.40)	2.83 (0.34)	3.06 (0.34)
HM	69	10.01%	3.99 (0.43)	4.00 (0.48)	3.89 (0.48)	2.93 (0.60)	3.22 (0.52)

Note: In Table 3, H/L represents the High Observation/Low Non-reactivity group, A/J represents the High Awareness/Judging group, MM represents the Moderately Mindful group, and HM represents the Highly Mindful group.

**Table 4:** Metacognitive score means and standard errors (M ± SE) for different mindfulness types among college students and the analysis of differences.

	P1	P2	P3	P4	BCH (*χ*^2^)	P1 VS. P2	P1 VS. P3	P1 VS. P4	P2 VS. P3	P2 VS. P4	P3 VS. P4
Planning	3.90(0.06)	3.50(0.07)	3.42(0.03)	4.27(0.07)	129.34 ***	19.67 ***	40.86 ***	14.92 ***	1.05	60.74 ***	108.52 ***
Monitoring	3.87(0.07)	3.36(0.06)	3.40(0.04)	4.27(0.08)	126.97 ***	29.18 ***	31.49 ***	15.08 ***	0.24	82.31 ***	99.72 ***
Adjustment	3.93(0.06)	3.59(0.06)	3.56(0.03)	4.33(0.07)	117.91 ***	17.02 ***	28.91 ***	19.90 ***	0.21	65.46 ***	101.88 ***
Evaluation	4.05(0.06)	3.59(0.06)	3.58(0.04)	4.39(0.07)	136.49 ***	27.36 ***	38.52 ***	13.82 ***	0.02	75.58 ***	110.89 ***

Note: *** *p* < 0.001.

## Data Availability

The datasets generated and analyzed during the current study are available from the corresponding author upon reasonable request.
